# Aqueous cytokine and growth factor levels do not reliably reflect those levels found in the vitreous

**Published:** 2011-11-09

**Authors:** Stephanie M. Ecker, Joshua C. Hines, Scott M. Pfahler, Bert M. Glaser

**Affiliations:** The National Retina Institute, Ocular Proteomics Laboratory, Towson, MD

## Abstract

**Purpose:**

Recent studies have illuminated the vitreous proteome as a potentially important diagnostic tool that will predict disease progression and response to treatment, in eyes with retinal disease. Studies to date have demonstrated correlations of protein levels between vitreous and aqueous humor. Because these results are un-expected and analysis was only done on a few endpoints, the present study further analyzes the relationship between aqueous and vitreous by probing a wide array of proteins in patients with posterior segment diseases.

**Methods:**

Anterior chamber aqueous fluid was obtained using a limbal approach with a 30 gauge needle. Immediately following, the vitreous sample was obtained via a pars plana approach. A 25 gauge needle with a 1 ml syringe was directed into the mid-vitreous cavity and vitreous fluid was gently aspirated. Aqueous and vitreous samples were then analyzed using the quantitative native protein analysis method called reverse phase protein microarray technology (RPPM).

**Results:**

The entire sample population (n=11) was probed against 34 proteins, revealing 8 proteins that significantly correlate, 3 proteins that trend to correlation but fell short of significance and 23 proteins that have no correlation between the vitreous and aqueous humor.

**Conclusions:**

Proteins in the aqueous cannot be assumed to correlate with their counterparts in the vitreous.

## Introduction

Recent reports have begun focusing on the presence and level of various proteins in intraocular fluid as markers of retinal disease [1-8]. This raises the opportunity to apply new technologies to study proteins found in intraocular fluid as markers for the identification, and prediction of treatment response in retinal disorders. One recent study demonstrated the ability to identify activated cell receptors and other proteins in vitreous aspirates, and found certain activated cell receptor proteins that correlate with response to bevacizumab treatment, suggesting that response to certain treatment options may be predicted by proteins in the vitreous [1].

The current school of thought is that aqueous samples are much easier to obtain from patients than a vitreous aspirate, because of past studies showing that the success rate of vitreous aspiration only around 35% [9]. However, our studies have shown that with proper technique, a 95% success rate can be achieved when performing in-office vitreous aspirations [10]. This led us to study the difference in vitreous and aqueous composition, to attempt to understand the differences and similarities of protein composition between the two chambers.

Although it is expected that the vitreous and aqueous would have different protein composition, previous studies on a small number of proteins have shown that the vitreous and aqueous protein levels do correlate [2,4,11]. Therefore, it is natural to ask about the relationship of protein levels in the vitreous and aqueous when considering optimal sampling strategies. This is especially important considering that there are different cell types populating the two chambers that are likely to contribute different proteins in addition to shared proteins.

The current study employs a reverse phase protein microarray (RPPM) based assay, which allows quantitative, high throughput, highly sensitive, simultaneous analysis of multiple proteins in their native state, using small sample volumes (50–100 μl) of either aqueous or vitreous [1,10,12,13], to discover the similarities and differences of the vitreous and aqueous humor proteomes.

## Methods

### Subjects

All enrolled patients completed informed consent for the surgical procedure and study, which was performed with full IRB approval (Western institutional review board, Olympia, WA) and adhered to the tenets of the Declaration of Helsinki. Every patient in the study had a vitreo-retinal condition (see Table 1) that required surgical intervention which was performed by the same surgeon (B.M.G.). Exclusion criteria included patients with any of the following: (1) History of cataract surgery, (2) active or previous intraocular inflammation, (3) anterior segment neovascularization or disease (conjunctival, corneal, iris, lens capsule) except for cataract, (4) retinal or ciliary body dialysis, or (6) unable to understand or complete informed consent.

**Table 1:** Patient population demographics.

**Patient number**	**Age**	**Diagnosis**	**Vitrectomy before surgery?**	**Eye**
Patient 1	65	Macular hole/Macular Pucker	No	OD
Patient 1*	65	Recurrent Macular Hole	Yes	OD
Patient 2	88	Retinal Detachment/Vitreous Membranes	Yes	OD
Patient 3	34	Retinal Detachment/Vitreous Membranes	No	OS
Patient 4	68	Macular hole/Macular Pucker	No	OS
Patient 5	71	Retinal Detachment	No	OD
Patient 6	67	Macular Hole	No	OD
Patient 7	72	Macular Hole	No	OS
Patient 8	50	Retinal Detachment	No	OS
Patient 9	69	Macular Hole	Yes	OS
Patient 10	90	Retinal Detachment	No	OS

Patient 1 had an initial sample taken before undergoing vitrectomy during macular hole surgery, Patient 1* represents the sample that was taken from this patient after a subsequent surgery to repair the recurrent macular hole.

### Sampling technique

A standard aqueous and vitreous sampling protocol was followed for all patients as follows: Following retrobulbar anesthesia, the patient was prepped and draped in a sterile fashion in the operating room. After a sterile lid speculum was placed, an infusion cannula was placed in the infratemporal quadrant for safety purposes. Once inserted, the infusion cannula remained in the off position until all sampling was completed. Anterior chamber aqueous fluid was obtained (0.05 ml) using a limbal approach with a 30 gauge needle (PrecisionGlide^®^; Becton Dickinson, Franklin Lakes NJ). Immediately following successful aqueous fluid sample, the vitreous sample was obtained via a pars plana approach. A 25 gauge needle (Terumo Needle; Terumo Corporation, Elkton, MD) with a 1 ml syringe (PrecisionGlide^®^; Becton Dickinson) was directed into the mid-vitreous cavity and 0.05 to 0.10 ml of vitreous fluid was gently aspirated. After removal, the syringe was capped, transferred to ice, and immediately transferred to a negative 80 °C freezer for storage. Once the aqueous/vitreous sampling was completed, the infusion line was inspected for proper placement and the remainder of the case was completed. No intravitreal drugs were administered during the course of this study to any of the patients.

### Proteomic analysis

A total of 34 proteins (Table 2) which represent endpoints from signaling pathways, such as angiogenesis, apoptosis, hypoxia/ischemia, oxidative stress, inflammation, and remodeling, all of which are known to be a part of vitreoretinal diseases [14-17] were selected to be studied. The selected proteins vary in size, confirmation, cellular localization, charge, and hydrophobicity. This amount of variation is used to ensure that there is no physical property or signaling pathway that specifically associates between the vitreous and aqueous, while others do not. The results reported here use this method to greatly expand the number of proteins that have been studied, which adds to the current knowledge base of biochemical reactions within the eye.

**Table 2:** List of antibodies and manufactures used in this study.

**Antibody**	**Primary pathway**	**Company**	**City, state**
αβ crystallin	Ocular Structure	Assay Designs	Plymouth Meeting, PA
AKT T308	Apoptosis/Survival	Cell Signaling	Danvers, MA
BAD S112	Apoptosis/Survival	Cell Signaling	Danvers, MA
BCL2 T56	Apoptosis/Survival	Cell Signaling	Danvers, MA
Musashi	Cell Death/Apop.	Cell Signaling	Danvers, MA
AMPK α1 S485	Hypoxia/Ischemia	Assay Designs	Plymouth Meeting, PA
cABL T735	Oxidative Stress	Cell Signaling	Danvers, MA
HemeOxygenase 1	Oxidative Stress	Assay Designs	Plymouth Meeting, PA
Integrin α5β1	Adhesion/Migration	Millipore	Billerica, MA
COX-2	Oxidative Stress	Invitrogen	Carlsbad, CA
C3a	Inflammation	Abcam	Cambridge, MA
C5a	Inflammation	Abcam	Cambridge, MA
C9	Inflammation	Abcam	Cambridge, MA
CF-H	Inflammation	Aby Shop	Gentofte, Denmark
IL-10	Inflammation	Abcam	Cambridge, MA
IL-12	Inflammation	Abcam	Cambridge, MA
IL-1β	Inflammation	Cell Signaling	Danvers, MA
IL-8	Inflammation	Abcam	Cambridge, MA
IL-6	Inflammation	Abcam	Cambridge, MA
TNF-α	Inflammation/Angio.	Cell Signaling	Danvers, MA
cKIT Y703	Angiogenesis	Invitrogen	Carlsbad, CA
cKIT Y719	Angiogenesis	Cell Signaling	Danvers, MA
FGFR Y653/654	Angiogenesis	Cell Signaling	Danvers, MA
PDGFRβ Y716	Angiogenesis	Millipore	Billerica, MA
PDGFRβ Y751	Angiogenesis	Cell Signaling	Danvers, MA
VEGF-A	Angiogenesis	Santa Cruz	Santa Cruz, CA
VEGFR2 Y1175	Angiogenesis	Cell Signaling	Danvers, MA
VEGFR2 Y951	Angiogenesis	Cell Signaling	Danvers, MA
VEGFR2 Y996	Angiogenesis	Cell Signaling	Danvers, MA
PEDF	Angiogenesis	Millipore	Billerica, MA
MMP-14	Remodeling/Angio.	Abcam	Cambridge, MA
MMP-2	Remodeling/Angio.	Cell Signaling	Danvers, MA
MMP-9	Remodeling/Angio.	Cell Signaling	Danvers, MA
TIMP2	Remodeling/Angio.	Abcam	Cambridge, MA

Vitreous and aqueous samples were analyzed by reverse phase protein microarray technology following the same protocol as described in [1]. Briefly, the samples were diluted in extraction buffer (T-PER [Pierce, Indianapolis, IN], 2-mercaptoethanol [Sigma, St Louis, MO], and 2× sodium dodecyl sulfate Tris-glycine loading buffer [Invitrogen, Carlsbad, CA]) prior to being plated in microtiter plate. The lysates were printed on glass-backed nitrocellulose array slides (FAST Slides; Whatman, Florham Park, NJ) using an Aushon 2470 arrayer (Aushon BioSystems, Burlington, MA) equipped with 350-μm pins. Each lysate was printed in a dilution curve representing undiluted and 1:2, 1:4, 1:8, and 1:16 dilutions. The slides were stored with desiccant (Drierite; WA Hammond, Xenia, OH) at –20 °C prior to immunostaining. Immunostaining was performed on an automated slide stainer, following the manufacturer’s instructions (Autostainer CSA kit; Dako, Fort Collins, CO) using polyclonal primary antibodies and biotinylated secondary antibodies.  Thirty four (34) primary antibodies were each incubated on their own slide at room temperature for 30 min, while two negative control slides incubated in the same manner with antibody diluents only on their own slides. All antibodies (Table 2) were previously validated in-house via western blot, and quality control RPPMs were printed with cellular controls and stained at multiple dilutions to determine the best signal/noise ratio. Each sample was printed onto 40 slides in duplicate along with standard cellular controls for the purpose of inter-slide precision, and data normalization. These controls include: A431+ EGF (BD Transduction Labs, San Jose, CA), A431 Cell lysate (BD Transduction Labs), BSA (ThermoScientific, Rockford, IL), and HeLa Pervanadate cells (BD Transduction Labs). Microarray spot analysis was completed in the same manner as described in [1,13].  Briefly, each array was scanned and spot intensity calculated using ImageQuant v5.2 (GE Healthcare, Piscataway, NJ), each spot duplicate was averaged and the data was normalized in Microsoft Excel (Microsoft, Redmond, WA) by subtracting the background noise from the negative slide and compared to one of the control cell lysates resulting in a single data point for each sample that could be compared to every other sample on the array.

### Statistical analysis

All statistical analysis was done using GraphPad Prism 5.0 (GraphPad Software, La Jolla, CA). The protein data was reported as relative intensity units as described in [12,13], a unit that expresses the quantitative abundance of the protein compared to background, and a paired t-test was used to compare the protein levels. To examine the correlations between the aqueous and vitreous, Shapiro-Wilk normality testing was performed for each group to determine the distribution of the data. Since the data are not normally distributed, the Spearman’s Rho correlation coefficient [18] was calculated for each protein. Probability values for the Spearman’s correlations were then calculated based on the t-distribution. A two-tailed p-value of less than 0.05 indicated statistical significance for both analyses. Ratio data was generated using the Bland-Altman comparison method comparing vitreous sample to matching aqueous sample; the resulting data are graphically represented. Further pathway analysis was performed using Pathway Studio (Ariadne Genomics, Rockville, MD) to understand how the proteins are interacting. All statistical work was reviewed and approved by the faculty at The Johns Hopkins Biostatistics Center (Baltimore, MD).

## Results

The study population included 10 patients with a total of 11 samples, which were all obtained in a surgical setting, with no intravitreal drug administered. One patient required a repeat operation for a chronic macular hole, and a second sample was obtained at this time (Patient 1*). Furthermore, two others (Patient 2 and 9) had vitrectomized eyes at the time of sample collection. The average time between prior vitrectomy and sampling for this study is 13 weeks, ranging from 3 weeks to 25 weeks. The patient population consisted of three male patients and seven female patients, while all involved eyes were phakic. The average age was 68.27 (range 34–90; See Table 1).

Levels of each protein in the aqueous and vitreous were quantified and a paired *t*-test and Spearman’s correlation coefficient were calculated. Aqueous and vitreous protein values were analyzed for the total study population (n=11).

### Vitreous and aqueous correlation analysis with all patients

The entire sample population was investigated via RPPM for 34 proteins. This process revealed eight proteins that significantly correlated between the vitreous and aqueous humor: AKT Threonine 308, (AKT T308; ρ=0.6727, p=0.0233), BCL-2 Associated Death Promoter Serine 112, (BAD S112; ρ=0.6727, p=0.0233), B cell lymphoma 2 Threonine 56, (BCL2 T56; ρ=0.6727, p=0.0233), Complement component 5a, (C5a; ρ=0.9091, p=0.0001), Complement component 9, (C9; ρ=0.6818, p=0.0208), Complement Factor H, (CF-H; ρ=0.9273, p=<0.0001), Fibroblast Growth Factor Receptor Tyrosine 653/654 (FGFR Y653/654; ρ=0.6545, p=0.0289), and Musashi (ρ=0.8273, p=0.0017).

Another subset of proteins trends toward correlation, but falls just short of being statistically significant. This subset includes: Cyclooxygenase 2 (COX-2; ρ=0.5909 p=0.0556), Interleukin-6 (IL-6; ρ=0.5989 p=0.0516), and Vascular Endothelial Growth Factor A (VEGFA; ρ=0.6000 p=0.0510). The remaining 23 screened proteins did not correlate in any significant way between the aqueous and the vitreous.

**Figure 1:**
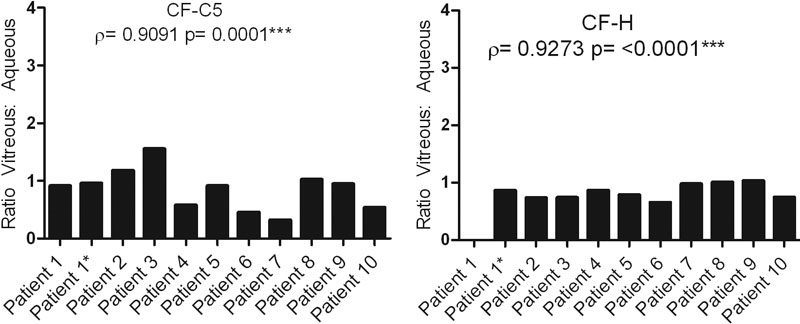
Demonstrates how the ratio of vitreous to aqueous protein levels is relatively constant for 2 proteins that showed a good correlation between the aqueous and vitreous. Patient 1 has no ratio level for CF-H because there was no detectable expression of this protein in the aqueous sample.

**Figure 2:**
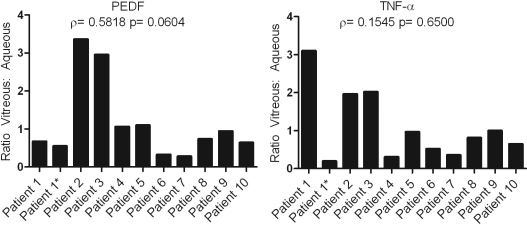
Graphical representation of the ratio of vitreous to aqueous levels of PEDF and TNF-α. These graphs display the marked difference in correlation of vitreous and aqueous protein levels from patient to patient.

To visualize this data, ratios of the vitreous versus the aqueous levels for each protein and in each patient combination were calculated and graphed. A true correlation between the vitreous and aqueous would demonstrate a constant ratio of level of expression between both compartments, and would be expected for all patients as can be seen in Figure 1 using CF-C5 and CF-H, which show a strong correlation in their levels between the vitreous and aqueous. In contrast, and representative of the majority of proteins studied, Figure 2 shows an example of TNF-α and PEDF where neither protein shows a significant correlation between the aqueous and vitreous. The variation in ratios from patient to patient exemplifies the protein level difference between the two chambers.

Protein network studies were done using Pathway Studio (Ariadne Genomics, Rockville, MD) to understand why the proteins that correlate between the aqueous and vitreous are correlating, and why the others are not. The proteins found to correlate between the aqueous and vitreous are a mainly part of two biologic groups, apoptosis, specifically negative regulation of apoptosis (GOgroup: 0043066) and alternative complement pathway activation (GOgroup: 0006957) and on a physical level, the 8 proteins that correlate are variable in size, charge and hydrophobicity. The proteins that do not correlate are not a part of either of these biologic processes, but are variable in their physical attributes. There is also a possibility that because of the different cellular composition around each of the chambers that the proteins produced are different, which leads to different interactions and binding partners for the proteins that are screened in this study. Still, we cannot definitively say that proteins that are a part of these pathways/processes would always track between the aqueous and vitreous.

To get a better understanding of the differences in protein interactions that occur in the aqueous and the vitreous, Spearman’s Rho analyses were performed (See Table 3). This data continues to show that the aqueous and vitreous have different proteomes.

**Table 3:** Pathway protein interactions that are exclusive to the aqueous or the vitreous.

**Vitreous**				**Aqueous**			
**Antibody**	**Antibody**	**Rho**	**p-value**	**Antibody**	**Antibody**	**Rho**	**p-value**
AKT T308	PDGFR Y716	0.855	2.20E-04	αB-Crystallin	AKT T308	0.875	4.00E-04
AKT T308	TIMP2	0.914	8.40E-05	αB-Crystallin	BAD Ser112	0.799	3.00E-03
AMPK a1 S485	BCL2 T56	0.888	0.001	αB-Crystallin	cKIT Y703	0.909	1.00E-04
AMPK a1 S485	COX2	0.802	0.003	αB-Crystallin	CF-H	0.763	6.00E-03
AMPK a1 S485	HemeOx-1	0.937	1.80E-05	αB-Crystallin	FGFR	0.911	1.00E-04
AMPK a1 S485	IL-8	0.943	1.40E-05	αB-Crystallin	MMP-2	0.89	2.00E-04
AMPK a1 S485	IL-10	0.878	3.70E-04	αB-Crystallin	PDGFR Y751	0.785	4.00E-03
AMPK a1 S485	MMP-9	0.82	2.00E-03	αB-Crystallin	VEGFR2 Y951	0.864	6.00E-04
AMPK a1 S485	Musashi	0.93	3.40E-05	BAD Ser112	FGFR	0.919	6.50E-05
BAD Ser112	COX2	0.873	5.00E-04	BCL2 T56	FGFR	0.919	6.50E-05
BAD Ser112	PDGFR Y716	0.808	8.00E-03	BCL2 T56	IL-12	0.782	4.00E-03
BAD Ser112	PDGFR Y751	0.75	3.00E-03	BCL2 T56	MMP-14	0.858	1.00E-03
BAD Ser112	TIMP2	0.794	4.00E-03	BCL2 T56	VEGFR2 Y951	0.87	4.90E-04
BAD Ser112	VEGFR2 Y996	0.857	1.00E-03	cABL T735	CF-H	0.801	3.00E-03
BCL2 T56	COX2	0.861	1.00E-03	cKIT Y719	Integrin a5b1	0.862	2.00E-03
BCL2 T56	IL-1B	0.821	2.00E-03	cKIT Y719	PDGFR Y716	0.764	6.00E-03
BCL2 T56	IL-8	0.782	4.00E-03	cKIT Y719	PDGFR Y751	0.823	2.00E-03
BCL2 T56	Musashi	0.759	7.00E-03	C3a	Integrin a5b1	0.8	3.00E-03
BCL2 T56	PDGFR Y716	0.782	4.00E-03	C5a	TNF alpha	0.801	3.00E-03
BCL2 T56	PDGFR Y751	0.786	6.00E-03	C5a	VEGFA	0.891	2.40E-04
BCL2 T56	VEGFR2 Y951	0.906	1.20E-04	C9	MMP-2	0.861	1.00E-03
BCL2 T56	VEGFR2 Y996	0.904	1.40E-04	C9	PDGFR Y716	0.793	4.00E-03
cABL T735	HemeOx-1	0.874	4.40E-04	C9	PDGFR Y751	0.784	4.00E-03
cABL T735	IL-1B	0.866	1.00E-03	C9	TIMP2	0.81	3.00E-03
cABL T735	IL-10	0.87	1.00E-03	FGFR	MMP-9	0.835	1.40E-03
cABL T735	PDGFR Y716	0.804	3.00E-03	FGFR	VEGFR2 Y951	0.814	2.30E-03
cABL T735	PEDF	0.782	4.00E-03	FGFR	VEGFR2 Y996	0.81	2.50E-03
cKIT Y703	COX2	0.862	1.00E-03	IL-1B	VEGFR2 Y951	0.762	6.00E-03
cKIT Y703	IL-1B	0.815	2.00E-03	Integrin a5b1	TNF alpha	0.801	3.00E-03
cKIT Y703	IL-10	0.762	6.00E-03	MMP-2	MMP-14	0.853	9.00E-04
cKIT Y703	MMP-14	0.86	1.00E-03	MMP-2	VEGFR2 Y951	0.82	2.00E-03
cKIT Y703	TIMP2	0.886	2.90E-04	MMP-9	MMP-14	0.862	1.00E-03
cKIT Y719	MMP-2	0.788	4.00E-03	MMP-14	VEGFR2 Y996	0.77	5.60E-03
COX2	MMP-9	0.888	2.60E-04	TNF alpha	VEGFA	0.893	1.00E-03
COX2	TIMP2	0.809	3.00E-03				
COX2	VEGFR2 Y951	0.823	2.00E-03				
HemeOx-1	IL-1B	0.875	4.20E-04				
HemeOx-1	IL-8	0.915	7.90E-05				
HemeOx-1	Musashi	0.896	1.90E-04				
HemeOx-1	TNF alpha	0.786	4.00E-03				
HemeOx-1	VEGFR2 Y996	0.847	1.00E-03				
HemeOx-1	VEGFR2 Y1175	0.823	2.00E-03				
IL-1B	IL-8	0.854	1.00E-03				
IL-1B	IL-10	0.932	2.90E-05				
IL-1B	Musashi	0.801	1.90E-04				
IL-8	IL-10	0.794	4.00E-03				
IL-8	Musashi	0.989	7.60E-09				
IL-8	VEGFR2 Y996	0.822	2.00E-03				
IL-10	Integrin a5b1	0.777	5.00E-03				
IL-10	MMP-2	0.889	1.00E-04				
IL-10	PDGFR Y716	0.932	2.90E-05				
IL-10	PDGFR Y751	0.839	1.00E-03				
IL-10	TNF alpha	0.819	2.00E-03				
IL-10	VEGFR2 Y996	0.911	9.60E-05				
Integrin a5b1	MMP-2	0.796	3.00E-03				
MMP-9	PDGFR Y751	0.788	4.00E-03				
MMP-14	TIMP2	0.89	2.40E-04				
MMP-14	VEGFR2 Y951	0.89	2.40E-04				
Musashi	VEGFR2 Y996	0.784	4.00E-03				
Musashi	VEGFR2 Y1175	0.773	5.00E-03				
PDGFR Y716	TIMP2	0.845	1.00E-03				
PDGFR Y716	TNF alpha	0.817	3.00E-03				
PDGFR Y751	TIMP2	0.896	1.90E-04				
PDGFR Y751	TNF alpha	0.809	3.00E-03				
PEDF	VEGFR2 Y1175	0.899	1.60E-04				
TIMP2	VEGFR2 Y951	0.802	3.00E-03				
TIMP2	VEGFR2 Y996	0.792	4.00E-03				
TNF alpha	VEGFR2 Y996	0.823	2.00E-03				

All of the samples that were taken post vitrectomy were compared to the group of samples that had no prior vitrectomy (Table 1). The post vitrectomy group was too small to make any significant conclusions, but investigating the pre-vitrectomy samples alone did demonstrate that the majority of the proteins screened still did not correlate between vitreous and aqueous samples.

**Figure 3:**
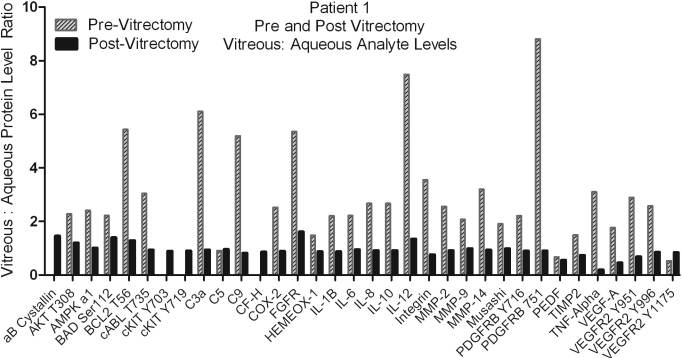
Represents the difference in the protein level ratios between aqueous and vitreous samples that were taken pre and post vitrectomy (Patient 1 and Patient 1*, respectively).

Additionally, Patient 1 had the first samples taken before any vitrectomy procedure had been performed, while the second sample set was taken 6 months after the patient had a vitrectomy. The ratios of vitreous to aqueous protein levels were graphed to visualize the difference in the correlation of the levels post vitrectomy (Figure 3). The ratios of the analyte levels between the vitreous and aqueous demonstrate more correlation between aqueous and vitreous after vitrectomy, yet there is still variation in the ratio from protein to protein. This data is very compelling, but must be studied in a broader group to see if this trend continues.

## Discussion

Mounting evidence from several studies to date have demonstrated that proteins in the vitreous correlate with occurrence and stage of various retinal disorders [1,3,5,7,19-21]. Such biomarkers could lead to improved understanding of retinal disease and enable the true realization of individualized treatment for a wide range of blinding retinal disorders.

**Figure 4:**
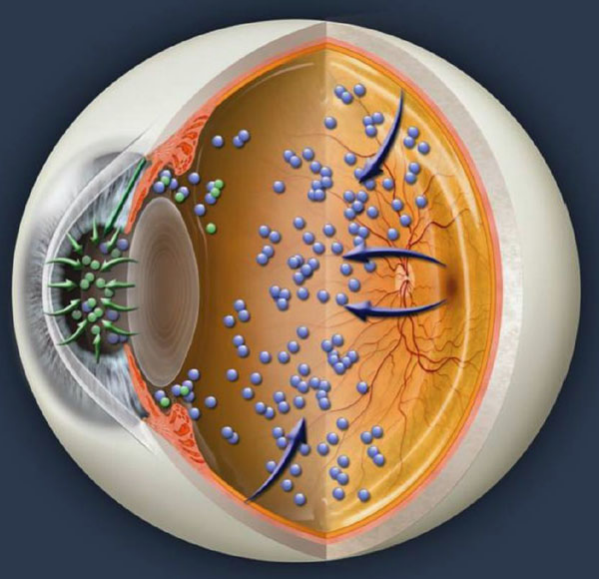
A cross-cut illustration demonstrating how proteins are likely to be produced in the aqueous and vitreous.

The vitreous and the aqueous are contained in two separate compartments that do not flow into one another. Although there may be some exchange of proteins between the chambers one cannot automatically conclude that all proteins present in the aqueous are derived from the retina, since the aqueous chamber is lined with its own unique cell types such as corneal endothelial cells, trabecular mesh work cells, lens cells, and iris cells (Figure 4). The vitreous chamber also has unique cell types including the cells of the retina, optic nerve, choroid, retinal pigment epithelium (RPE cells) and at times, newly formed vitreous blood vessels (Figure 4). When analyzing similarities and differences between the two compartments, various factors such as lens status, vitreous structure, disease state, and protein size must be considered. Differing rates of protein synthesis and metabolism between the aqueous and vitreous could produce differences in the proteome of each respective segment. The differences in tissue type between the two compartments may also contribute to a quantitative difference in levels of various proteins.

In disease states localized to the retina and choroid such as macular degeneration, protein levels in the vitreous may be a better indicator than protein levels in the aqueous. Studying the pathobiology of vitreoretinal diseases would seemingly be most effective by probing the vitreous because of its close proximity to the retina, and thus a better sample to interrogate for biomarkers of retinal diseases or clues toward new retinal disease treatments.

Of the thirty-four (34) proteins analyzed, statistically significant correlations between aqueous and vitreous levels occurred in only eight (8) proteins and strong (yet not statistically significant) trends could be seen in only three (3) additional proteins. The 8 correlating proteins are representative of the apoptosis and complement activation protein families, but there is no known reason to assume why these particular proteins would correlate, whereas the other 24 do not.

Past reports have analyzed VEGF levels in both compartments and hypothesized that anterior segment levels reflect the levels in the vitreous [6]. This hypothesis has been supported by a publication reporting corresponding aqueous and vitreous levels of VEGF and IL-6 in diabetic retinopathy patients [4]. Even though this study was done on a small set of heterogeneous patients, we do achieve statistically significant data, and with our ability to do this in 34 proteins, the work presented expands on the past studies regarding the correlation of levels of VEGF-A and IL-6 between aqueous and vitreous samples in the same patient [4,11]. Expanding the number of investigated proteins shows that not all proteins track each other between the aqueous and vitreous. These results show a positively trending correlation (short of statistically significant) for the same two (2) proteins (VEGF-A and IL-6), however, this study found absolutely no correlation in 24 of 34 total proteins. To the best of this group’s knowledge, no one has reported extensive correlating protein levels measured in the vitreous and the aqueous of humans or in animal studies. Further studies on the correlation of protein markers between the aqueous and vitreous in other diseases and in “normal” samples would be ideal to learn how the interaction of proteins between the bodies changes depending on diseases state, unfortunately this type of study could not be performed due to the unnecessary invasive procedures that would be done on healthy individuals, but animal studies could be developed to understand the correlation further.

The work presented here does support the previous studies comparing the aqueous and vitreous, but they only studied 2 proteins, and were unable to demonstrate how generalized the phenomena is. By expanding the number of proteins studied from 2 to 34, we were able to show that the majority of the protein levels in the aqueous do not reflect those in the vitreous. As a result of these findings, the reliability of aqueous protein levels reflecting vitreous protein levels must be tested for each protein biomarker of interest.
